# A clinical variable‐based nomogram could predict the survival for advanced NSCLC patients receiving second‐line atezolizumab

**DOI:** 10.1002/cam4.4160

**Published:** 2021-07-31

**Authors:** Xiaoling Shang, Jianxiang Shi, Xiaohui Wang, Chenglong Zhao, Haining Yu, Haiyong Wang

**Affiliations:** ^1^ Department of Clinical Laboratory Shandong University Jinan China; ^2^ Department of Clinical Laboratory Shandong Cancer Hospital and Institute Shandong First Medical University and Shandong Academy of Medical Sciences Jinan China; ^3^ Henan Academy of Medical and Pharmaceutical Sciences Zhengzhou University Zhengzhou China; ^4^ Precision Medicine Center Zhengzhou University Zhengzhou China; ^5^ Research Service Office Shandong Liaocheng People’s Hospital Liaocheng China; ^6^ Department of Pathology Shandong Cancer Hospital and Institute Shandong First Medical University and Shandong Academy of Medical Sciences Jinan China; ^7^ Personnel Division Shandong Cancer Hospital and Institute Shandong First Medical University and Shandong Academy of Medical Sciences Jinan China; ^8^ Department of Internal Medicine‐Oncology Shandong Cancer Hospital and Institute Shandong First Medical University and Shandong Academy of Medical Sciences Jinan China

**Keywords:** atezolizumab, docetaxel, nomogram, NSCLC, overall survival

## Abstract

**Objective:**

A nomogram model based on clinical variables was conducted to predict the survival in patients with non‐small cell lung cancer (NSCLC) receiving second‐line atezolizumab.

**Methods:**

Four hundred and twenty‐four patients with NSCLC receiving atezolizumab from OAK study were regarded as the training cohort. Next, a nomogram model based on clinical variables in the training cohort was established to predict the survival of patients receiving atezolizumab. The concordance index, area under curve (AUC), and calibration plots were used to assess the performance of the nomogram model. In addition, 144 patients with NSCLC receiving atezolizumab from POPLAR study were regarded as the test cohort to validate the nomogram model. Using Kaplan–Meier and log‐rank test, we compared the survival difference between the high‐ and low‐risk groups, atezolizumab and docetaxel treatment groups, respectively.

**Results:**

We successfully constructed a nomogram model based on different variable scores for predicting the survival in NSCLC patients receiving atezolizumab using the training cohort. According to risk score, patients receiving atezolizumab were divided into the high‐ and low‐risk groups. Importantly, in the training cohort, patients had worse overall survival (OS) in high‐risk group compared with the low‐risk group (median survival: 252.3 vs. 556.9 days; *p* < 0.0001). As expected, in the test cohort, the high‐risk patients also showed a worse OS (median survival: 288.8 vs. 529.3 days, *p* = 0.0003). In addition, all the patients from the training and test cohorts could be found the OS benefit from atezolizumab compared with docetaxel (all, *p* < 0.05).

**Conclusions:**

The clinical variable‐based nomogram model could predict the survival benefit for NSCLC patients receiving second‐line atezolizumab.

## Graphical abstract

The clinical variable‐based nomogram model could predict the survival in patients with NSCLC receiving second‐line atezolizumab.

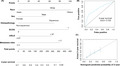

## 1 INTRODUCTION

Non‐small cell lung cancer (NSCLC) represents a massive health burden worldwide, which is the main cause of cancer death.^1^ Immunotherapy has become the latest milestone in the treatment of metastatic and advanced NSCLC population, especially the application of immune checkpoint inhibitors (ICIs).^2^, ^3^, ^4^ The use of programmed cell death‐1 (PD‐1) inhibitors or programmed death‐ligand 1 (PD‐L1) inhibitors is successful in achieving improved prognosis of advanced NSCLC patients either in first‐ or second‐line settings in clinical trials.^5^, ^6^, ^7^, ^8^, ^9^ However, not all advanced or metastatic patients with NSCLC could benefit from PD‐1/PD‐L1 inhibitors, suggesting the urgent need to select the right candidates.^10^, ^11^

Increasing evidence has demonstrated that tumor mutational burden (TMB) and the expression of PD‐L1 could be used as biomarkers to select those patients who would benefit from ICIs.^12^, ^13^, ^14^ However, TMB could not differentiate the overall survival (OS) of patients benefits after immunotherapy treatment,^8^, ^9^, ^15^ leading the clinical utility of TMB as an actionable predictor of immunotherapy and remains controversial which has attracted clinicians’ widespread attention.^16^, ^17^ In addition, the detection of PD‐L1 expression requires a tissue sample and could not be sufficiently representative of overall tumor/metastasis expression, impeding treatment decision‐making. Moreover, dynamic changes of PD‐L1 expression in tumor cells might occur before or during treatment of ICIs, which lead to different sensitivity to ICIs agents and would be missed by a single biopsy. All above problems may limit the clinical application of TMB and PD‐L1 expression detection to predict the outcomes for those patients with NSCLC who receiving ICIs treatment.

Additional promising blood‐ and tissue‐based biomarkers are under investigation, such as T‐cell receptor clonality, tumor‐infiltrating lymphocytes, mutational or neoantigen burden, lymphocyte, neutrophil counts, the ratio of granulocyte to lymphocyte, immune gene signatures, MHC status, microbiome profile, and so forth.^18^ However, these biomarkers are under investigation and the detection efficiency remains to be explored.

Among clinical factors, race was reported to be related to the efficacy of ICIs treatment for patients with advanced NSCLC.^19^ In a previous meta‐analysis, Fabio Conforti, et al. demonstrated that there was significant different efficacy between male and female in the treatment of ICIs.^20^ Furthermore, in patients with NSCLC receiving ICIs treatment, the involvement of more than one metastatic site and Eastern Cooperative Oncology Group performance status (ECOG PS) were independently related to shorter OS.^21^

Therefore, in the present study, we would like to construct a nomogram model based on clinical variables to predict the survival in patients with NSCLC treated with atezolizumab.

## 2 MATERIALS AND METHODS

### 2.1 Patients

Data of our present study were obtained from a previous study by Gandara et al.^22^ Using a retrospective analysis of OAK and POPLAR trials, the study showed that high blood‐based tumor mutational burden (bTMB) was a clinically actionable biomarker for atezolizumab in patients with NSCLC. Based on the published database, we performed a second analysis and developed a nomogram model with clinical variables to predict the survival in patients with NSCLC receiving second‐line atezolizumab treatment. In the OAK study, a total of 425 patients receiving atezolizumab were included in this study. One patient was excluded due to the lack of the specific information of baseline sum of the longest diameters (blSLD). Finally, among them, 424 patients were included in the training cohort and used to build a nomogram model based on clinical variables. In the test group, 144 NSCLC patients receiving atezolizumab from the POPLAR trial were applied to make a validation. Next, another 425 patients with NSCLC receiving docetaxel from the OAK cohort and 143 NSCLC patients treated with docetaxel from the POPLAR cohort were also included in this study. As all data were obtained from the published database, the informed consent and ethics committee approval were not required.

### 2.2 Statistical analysis

The χ^2^ test was used to analyze differences in clinicopathologic variables between the training group and the test group. The univariate and multivariate Cox regression analyses were used to analyze the impact of clinicopathological variables on survival. Multivariate Cox proportional hazard regression was used to analyze the prognostic clinical factors that were considered as potential correlation in univariate analysis. And then, according to the variables that remained statistically significant (*p* < 0.05) in multivariate analysis, a nomogram was established to predict the 2‐year survival rate for patients with NSCLC receiving atezolizumab. Harrell's concordance index (C‐index) was measured to quantify the discrimination ability of the nomogram, while the receiver operating characteristic (ROC) curve was used to determine the sensitivity, specificity and area under curve (AUC) was calculated to evaluate the diagnostic efficiency of the nomogram model. Similar to AUC, the C‐index was a generalization of AUC that could consider censored data. C‐index = 0.5 represented a random prediction while C‐index = 1 corresponded to the best model prediction. Next, the 2‐year survival rate was calibrated by calibration curve after comparing the actual survival rate with the predicted probability of survival by nomogram. Finally, according to the risk score, all patients were classified into the low‐ and high‐risk groups. The Kaplan–Meier method was used to analyze the patients’ OS and progression‐free survival (PFS) and the *p* value was determined by log‐rank test. All statistical analyses were made using R‐package software and Statistical Product Service Solutions (SPSS) 22.0 software. A *p* less than 0.05 was considered to be statistically significant.

## 3 RESULTS

### 3.1 Patients characteristics

A total of 424 patients with NSCLC treated with atezolizumab were included in the training cohort. Furthermore, another 144 patients receiving atezolizumab were included in the test cohort. Overall, in the training and the test cohort, the proportion of patients aged less than 65 years old (58.0 and 65.3%) was the largest. Most of patients were white (71.3 and 76.4%) and male (61.3 and 64.6%). Moreover, patients with non‐squamous NSCLC accounted for 73.8% in the training cohort and 66.0% in the test cohort. There were apparent disparities between the groups of ECOG PS, previous treatment, tobacco history, and number of metastasis sites. Most of patients in the training cohort and the test chort (63.4 and 66.7%) had ECOG PS of 1. The proportion of previous/current smokers (80.2 and 81.2%) was greater in training cohort and test cohort compared with never smokers (19.8 and 18.8%). According to the median of blSLD was 67 mm, patients were classified into two groups. Patients in the two groups accounted for about the same proportion (50.5 vs. 49.5% and 45.1 vs. 54.9%). A total of 70.5% patients in the training cohort and 71.5% patients in the test cohort had less than three metastasis sites. Except for previous treatment (*p* = 0.013), there was no significant difference in other clinical variables between two groups (all, *p* > 0.05). The specific baseline clinicopathological variables were reported in Table 1.

**TABLE 1:** Baseline clinicopathological characteristics of all patients with NSCLC treated with atezolizumab in the training cohort and the test cohort

Variables	Training cohort (*n* = 424)	Test cohort (*n* = 144)	*p*
Age			0.125
≤65	246 (58.0)	94 (65.3)	
>65	178 (42.0)	50 (34.7)	
Race			0.476
White	302 (71.3)	110 (76.4)	
Asian	85 (20.0)	23 (16.0)	
Others	37 (8.7)	11 (7.6)	
Sex			0.486
Male	260 (61.3)	93 (64.6)	
Female	164 (38.7)	51 (35.4)	
Histology			0.070
Non‐squamous	313 (73.8)	95 (66.0)	
Squamous	111 (26.2)	49 (34.0)	
ECOG PS			0.486
0	155 (36.6)	48 (33.3)	
1	269 (63.4)	96 (66.7)	
Previous treatment			0.013
1	319 (75.2)	93 (64.6)	
2	105 (24.8)	51 (35.4)	
Smoking status			0.781
Never	84 (19.8)	27 (18.8)	
Previous/Current	340 (80.2)	117 (81.2)	
blSLD (mm)			0.269
≤67	214 (50.5)	65 (45.1)	
>67	210 (49.5)	79 (54.9)	
Metastatic sites			0.818
1–3	299 (70.5)	103 (71.5)	
>3	125 (29.5)	41 (28.5)	

blSLD, baseline sum of the longest diameters; ECOG, Eastern Cooperative Oncology Group; PS, performance status.

### 3.2 Prognostic factor analysis of OS

In the training cohort, the result of univariate analysis revealed that race (*p* = 0.0449), sex (*p* = 0.006), histology (*p* = 0.002), ECOG PS (*p* < 0.001), blSLD (*p* < 0.001), and number of metastasis sites (*p* < 0.001) were associated with the OS in patients treated with atezolizumab. However, age, previous treatment, and smoking status were not related to the OS (all, *p* > 0.05). In the multivariate analysis, the result revealed that race (*p* = 0.026), sex (*p* = 0.032), histology (*p* = 0.006), ECOG PS (*p* < 0.001), blSLD (*p* = 0.0049), and number of metastasis sites (*p* < 0.001) were significant independent prognostic factors and were further incorporated into the predictive nomogram model. The univariate and multivariate analyses were listed in Table 2.

**TABLE 2:** Univariate and multivariate analyses of each factor's ability in predicting the OS for 424 patients with NSCLC receiving atezolizumab from OAK cohort in the training cohort

	Univariate analysis			Multivariate analysis		
Variables	HR	95% CI	*p*	HR	95% CI	*p*
Age				NI		
≤65	Reference					
>65	0.993	0.7805–1.263	0.954			
Race						
White	Reference			Reference		
Asian	0.716	0.517–0.993	0.0449	0.689	0.496–0.957	0.026
Others	1.152	0.771–1.724	0.490	1.207	0.804–1.811	0.364
Sex						
Male	Reference			Reference		
Female	0.705	0.548–0.906	0.006	0.755	0.585–0.976	0.032
Histology						
Squamous	Reference			Reference		
Non‐Squamous	0.665	0.512–0.863	0.002	0.683	0.520–0.897	0.006
ECOG PS						
0	Reference			Reference		
1	1.725	1.334–2.231	< 0.001	1.715	1.323–2.223	< 0.001
Previous treatment				NI		
1	Reference					
2	0.938	0.713–1.235	0.648			
Smoking status				NI		
Previous	Reference					
Current	0.738	0.512–1.063	0.102			
Never	0.768	0.558–1.057	0.106			
blSLD						
≤67	Reference			Reference		
>67	1.648	1.296–2.096	< 0.001	1.426	1.114–1.826	0.0049
Metastatic site						
1–3	Reference			Reference		
>3	1.591	1.241–2.039	< 0.001	1.563	1.203–2.030	< 0.001

blSLD, baseline sum of the longest diameters; ECOG, Eastern Cooperative Oncology Group; NI, not included; PS, performance status.

### 3.3 Construction of a nomogram model to predict the survival

**FIGURE 1:**
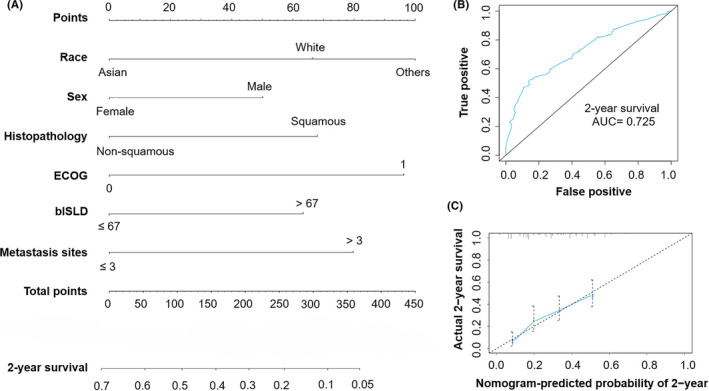
The development of a nomogram model to predict the overall survival (OS) NSCLC patients receiving atezolizumab from the OAK cohort. (A) Prognostic nomogram for NSCLC patients to assign their probability of survival at 2‐year after atezolizumab treatment. (B) The area under the curve (AUC) of the prognostic nomogram was 0.725. C: Calibration curve of the nomogram predicting 2‐year OS rate of NSCLC patients receiving atezolizumab treatment. On the calibration plot, the x‐axis was nomogram‐predicted probability of 2‐year survival. The y‐axis was actual 2‐year survival

According to the regression coefficients estimated in multivariate analysis, a prognostic nomogram model based on clinical variables including race, sex, histology, ECOG PS, blSLD, and number of metastasis sites was established to determine the 2‐year survival probability after atezolizumab treatment commencement (Figure 1A). The C‐index of OS model was 0.634. And from Figure 1B, the ROC curve showed that the AUC in the training cohort was 0.725. In fact, the prediction will fall on the 45‐degree diagonal line in a well‐calibrated model. Figure 1C revealed that calibration of the nomogram for the OS was well adequate.

### 3.4 The influence of risk group on OS based on the nomogram model

According to the total scores of each patient in the training cohort, we developed a risk classification system. All patients with NSCLC receiving atezolizumab were classified into the high‐ and low‐risk groups based on the nomogram model risk score. The optimal cutoff value of the nomogram model risk score was 1. Risk score greater than 1 was included in high‐risk group, and risk score less than 1 was incorporated into low‐risk group.

**FIGURE 2:**
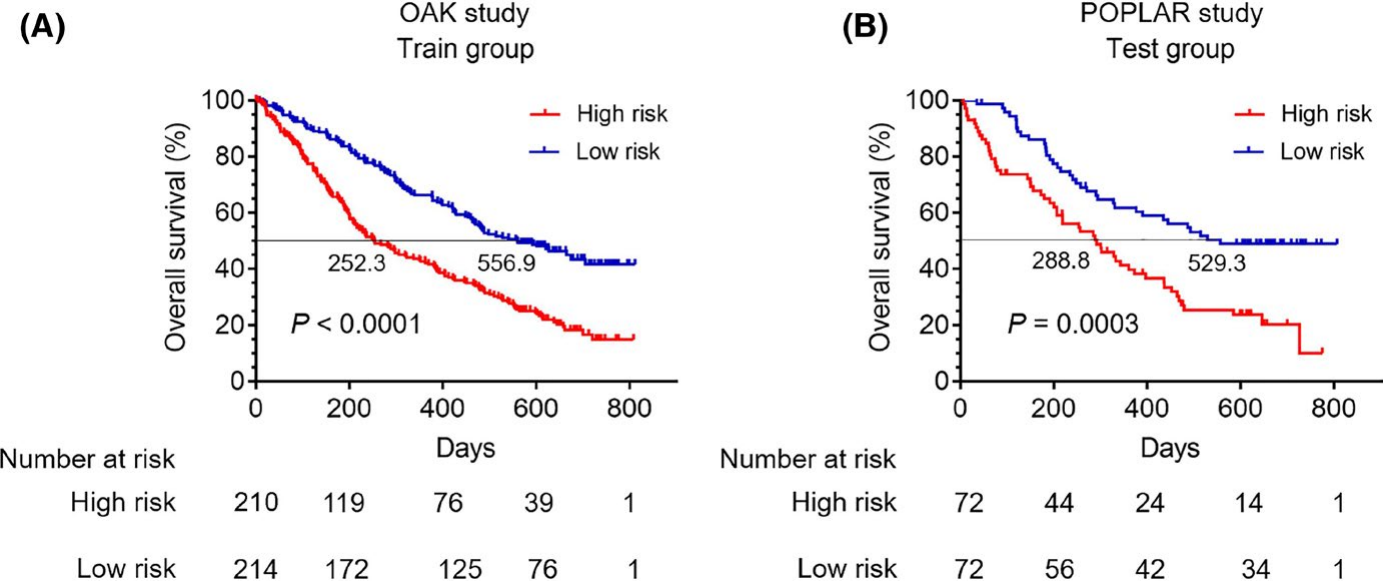
The overall survival (OS) comparison for NSCLC patients receiving atezolizumab treatment in the low‐ and high‐risk groups. (A) Survival comparison for NSCLC patients in the low‐ and high‐risk groups of training cohort from the OAK cohort (*p* < 0.0001) (Median OS: 556.9 vs. 252.3 days). (B) Survival comparison for patients in the low‐ and high‐risk groups of validation cohort from the POPLAR cohort (*p* = 0.0003) (Median OS: 529.3 vs. 288.8 days)

In the training cohort, patients’ baseline characteristics in the high‐ and low‐risk groups were shown in Table S1. Survival curves revealed that patients in the high‐risk group had significantly worse OS compared with those in the low‐risk group (*p* < 0.0001) (Median OS: 252.3 vs. 556.9 days) (Figure 2A). Similarly, patients in high‐risk group had worse progression‐free survival (PFS) than those in low‐risk group (*p* = 0.0062) (Figure S2A). Furthermore, the test cohort was applied to validate the nomogram model. The ROC curve showed that the AUC of the test cohort was 0.58 (Figure S1A). And the calibration of the nomogram model for the OS in the test cohort was also well adequate (Figure S1B). In the test cohort, patients’ baseline characteristics in the high‐ and low‐risk groups were listed in Table S2. As expected, survival curves revealed that the OS was also significantly worse for patients in the high‐risk group compared with those in the low‐risk group (*p* = 0.0003) (Median OS: 288.8 vs. 529.3 days) (Figure 2B) and patients in the high‐risk group had worse PFS than those in the low‐risk group (*p* = 0.0205) (Figure S2B).

### 3.5 Survival comparison of all patients treated with atezolizumab and docetaxel

**FIGURE 3:**
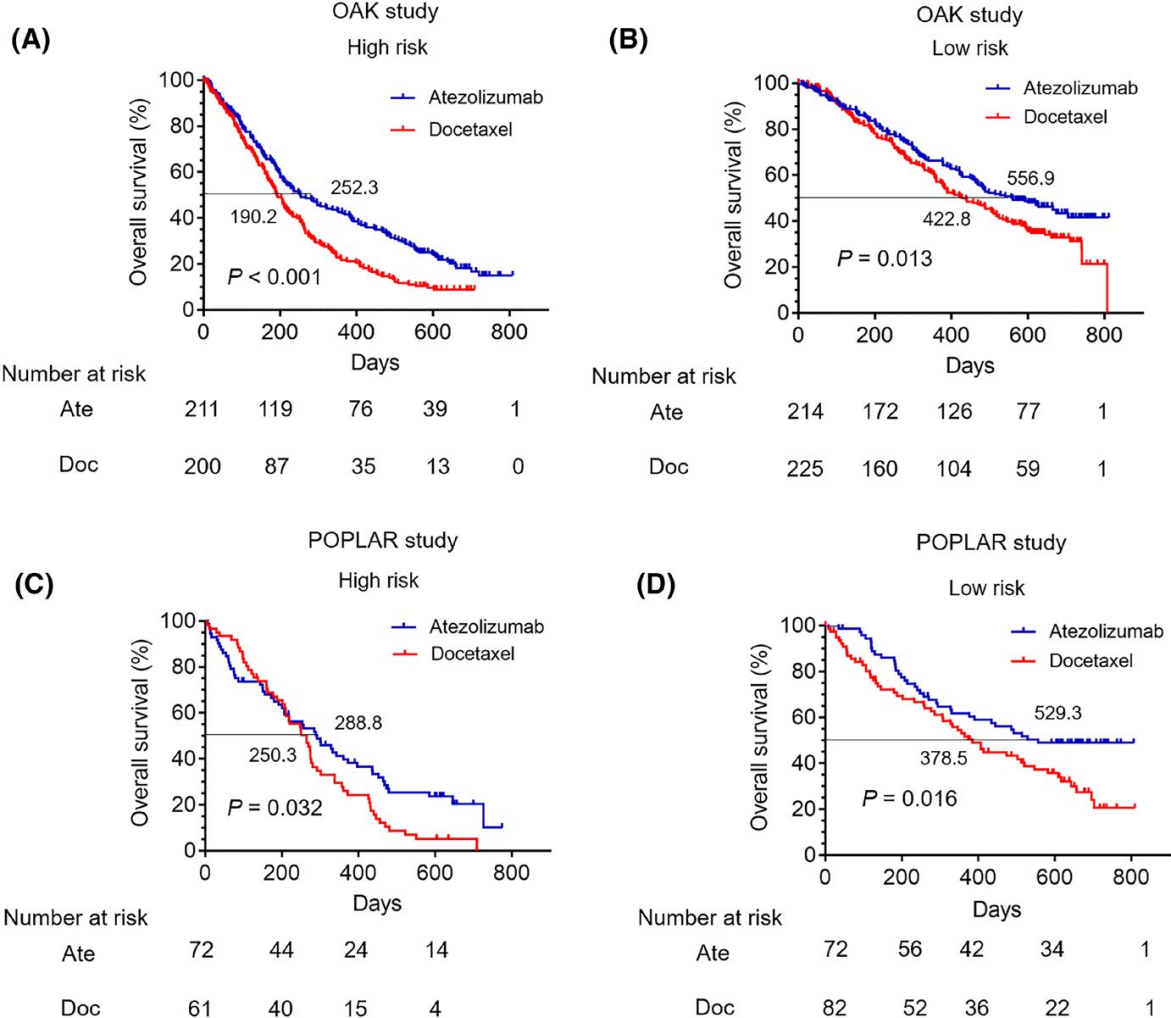
The overall survival (OS) comparison for NSCLC patients between receiving atezolizumab and docetaxel treatment in the low‐ and high‐risk groups. (A) Survival comparison for NSCLC patients between receiving atezolizumab and docetaxel treatment in the high‐risk group from the OAK cohort (*p* < 0.001) (Median OS: 252.3 vs. 190.2 days). (B) Survival comparison for NSCLC patients between receiving atezolizumab and docetaxel treatment in the low‐risk group from the OAK cohort (*p* = 0.013) (Median OS: 556.9 vs. 422.8 days). (C) Survival comparison for NSCLC patients between receiving atezolizumab and docetaxel treatment in the high‐risk group from the POPLAR cohort (*p* = 0.032) (Median OS: 288.8 vs. 250.3 days). (D) Survival comparison for NSCLC patients between receiving atezolizumab and docetaxel treatment in the low‐risk group from the POPLAR cohort (*p* = 0.016) (Median OS: 529.3 vs. 378.5 days)

In order to compare survival differences of patients receiving atezolizumab and docetaxel in the high‐ or low‐risk group, respectively, a total of 425 NSCLC patients treated with docetaxel from the OAK cohort were included in this study. Then, according to the risk score, all patients were classified into the high‐ and low‐risk groups (Table S3). Survival curves revealed that patients receiving atezolizumab had better OS than those receiving docetaxel in the high‐risk group (*p* < 0.001) (Median OS: 252.3 vs. 190.2 days) (Figure 3A), while there was no significant difference in PFS between patients receiving atezolizumab and docetaxel (*p* = 0.1580) (Figure S3A). In the low‐risk group, the OS in patients with atezolizumab monotherapy was longer than those with docetaxel therapy (*p* = 0.013) (Median OS: 556.9 vs. 422.8 days) (Figure 3B), while the PFS showed no significant difference (*p* = 0.7095) (Figure S3B).

Furthermore, 143 patients receiving docetaxel treatment from the POPLAR trial were also included in this study. The baseline clinicopathological characteristics of patients treated with docetaxel of the POPLAR cohort in the high‐ and low‐risk groups were reported in Table S4. In the high‐risk group, patients receiving atezolizumab had a relative better OS than those receiving docetaxel (*p* = 0.032) (Median OS: 288.8 vs. 250.3 days) (Figure 3C), while there was no significant difference in PFS between the two groups (*p* = 0.5365) (Figure S3C). Meanwhile, in the low‐risk group, the OS in patients receiving atezolizumab was relatively longer than docetaxel (*p* = 0.016) (Median OS: 529.3 vs. 378.5 days) (Figure 3D), while the PFS showed no significant difference (*p* = 0.4195) (Figure S3D).

## 4 DISCUSSION

In the past few years, an unprecedented number of immunotherapeutic agents such as anti‐PD‐L1, atezolizumab, have been proved the better survival benefit compared with standard chemotherapy in advanced patients with NSCLC, which greatly has expanded treatment selections beyond first‐line treatment. However, in spite of a proportion of patients achieving a long‐term disease control, about 60–80% of patients progress on ICIs carrying a dismal outcome.^23^ Therefore, it is an urgent unmet need to properly select the right candidates to receive immunotherapy.

Of interest, some of research proposed clinical prognostic parameters for atezolizumab in the treatment of advanced patients with NSCLC, among which was race.^19^ Our result revealed that race was a prognostic factor of survival in patients with NSCLC receiving atezolizumab treatment, which was consistent with previously described findings in the literature.^19^ Furthermore, it was reported that the difference in efficacy between male and female treated with ICIs including ipilimumab, tremelimumab, nivolumab, and pembrolizumab was significant.^20^ More importantly, our results revealed that sex was also a significant independent prognostic factor for atezolizumab in the treatment of advanced NSCLC patients. In the training cohort, among disease characteristics, univariate and multivariate analyses also identified histopathology, ECOG PS, blSLD, and number of metastatic sites as risk factors related to the OS in patients treated with atezolizumab. We found that patients with squamous cell lung cancer, higher PS, larger blSLD (> 67 mm), and more than three metastatic sites had worse OS. These findings were consistent with the results by Qian et al.^19^

In the current study, we assessed the role of clinical features in order to establish a nomogram model in patients with advanced NSCLC receiving atezolizumab as second‐line treatment to enable individual OS estimation. The prognostic nomogram model was based on readily available, inexpensive, and easy to collect patients (race, sex, and ECOG PS) and disease variables (histopathology, blSLD, and number of metastatic sites). Our results demonstrated that patients receiving atezolizumab with ECOG PS of 1 had poorer outcome than those with ECOG PS of 0. Notably, patients with ECOG PS of 2 or higher were usually excluded from clinical trials assessing the efficacy of ICIs. In fact, more than 20% of such patients received ICIs treatment in real‐world practice. However, due to the limitation of public database, our study did not involved those patients with ECOG PS of 2 or higher which may lead some result biases.

Moreover, blSLD was another important predictive factor, which was commonly taken into consideration in routine clinical practice. Our result was similar to the finding by previous research which demonstrated that blSLD was associated with the OS in advanced NSCLC patients receiving atezolizumab treatment. In another study, baseline tumor size was indicated as a prognostic factor of the OS in advanced melanoma patients treated with pembrolizumab.^24^ Therefore, blSLD should be routinely measured to strengthen its potential use as a prognostic predictor in clinical practice.

As demonstrated in a previous meta‐analysis, the combined predictive utility of PD‐L1 expression and TMB was associated predictive prognosis and was usually used as biomarkers of first‐ or second‐line immunotherapy in patients with NSCLC.^25^ In clinical practice, the utility of these biomarkers was limited because patients who did not express PD‐L1 may respond to ICIs, while some patients with elevated PD‐L1 expression may not benefit from these drugs.^26^ In the present study, we established a clinical variable‐based nomogram model as a marker to select candidates to receive second‐line atezolizumab monotherapy. The predictive tool could be available to make treatment decision for clinicians.

In our study, the 2‐year survival rate was defined as our endpoint. The nomogram performed well by AUC, which showed that our nomogram had a good performance to predict the 2‐year OS rate for NSCLC patients treated with atezolizumab. Meanwhile, the calibration curve revealed good consistent between nomogram‐predicted probability of 2‐year survival and actual observed 2‐year survival. To apply the nomogram, a vertical line should be delineated to the point row to assign point values for each variable. Next, the corresponding points are summed to get the total points. Finally, a vertical line from the total points needs to be drawn to gain the value of 2‐year OS probability.

However, some limitations in this study should be noted. Firstly, this is a respective research from the public database. Secondly, due to the limitation of public database, some important immune‐related indicators such as CD4+ T cell, CD8+ T cell, and other clinical parameters were not included in this study, all of which may affect the survival prediction for patients receiving immunotherapy. Thirdly, due to the patients with ECOG PS of 2 or higher were excluded by the clinical trials, we could not gain the association between them with survival in the real‐world practice.

## 5 CONCLUSION

In the present study, we built an inexpensive and easy‐to‐use tool to assist clinicians with a quantitative mean to predict the survival in advanced NSCLC treated with atezolizumab in clinical practice. The predictive tool could be available to make treatment decision for clinicians.

## CONFLICT OF INTEREST

All authors declared that they have no conflict of interest.

## Supporting information

Fig S1

Fig S2

Fig S3

Data S1

## Data Availability

The data that support the findings of this study are available from the corresponding author upon reasonable request.
