# Supplementary figures and images for: A clinical variable‐based nomogram could predict the survival for advanced NSCLC patients receiving second‐line atezolizumab
Source: Cancer Med. 2021 Jul 31;10(18):6218–26. doi: 10.1002/cam4.4160 (PMC8446569; doi:10.1002/cam4.4160)

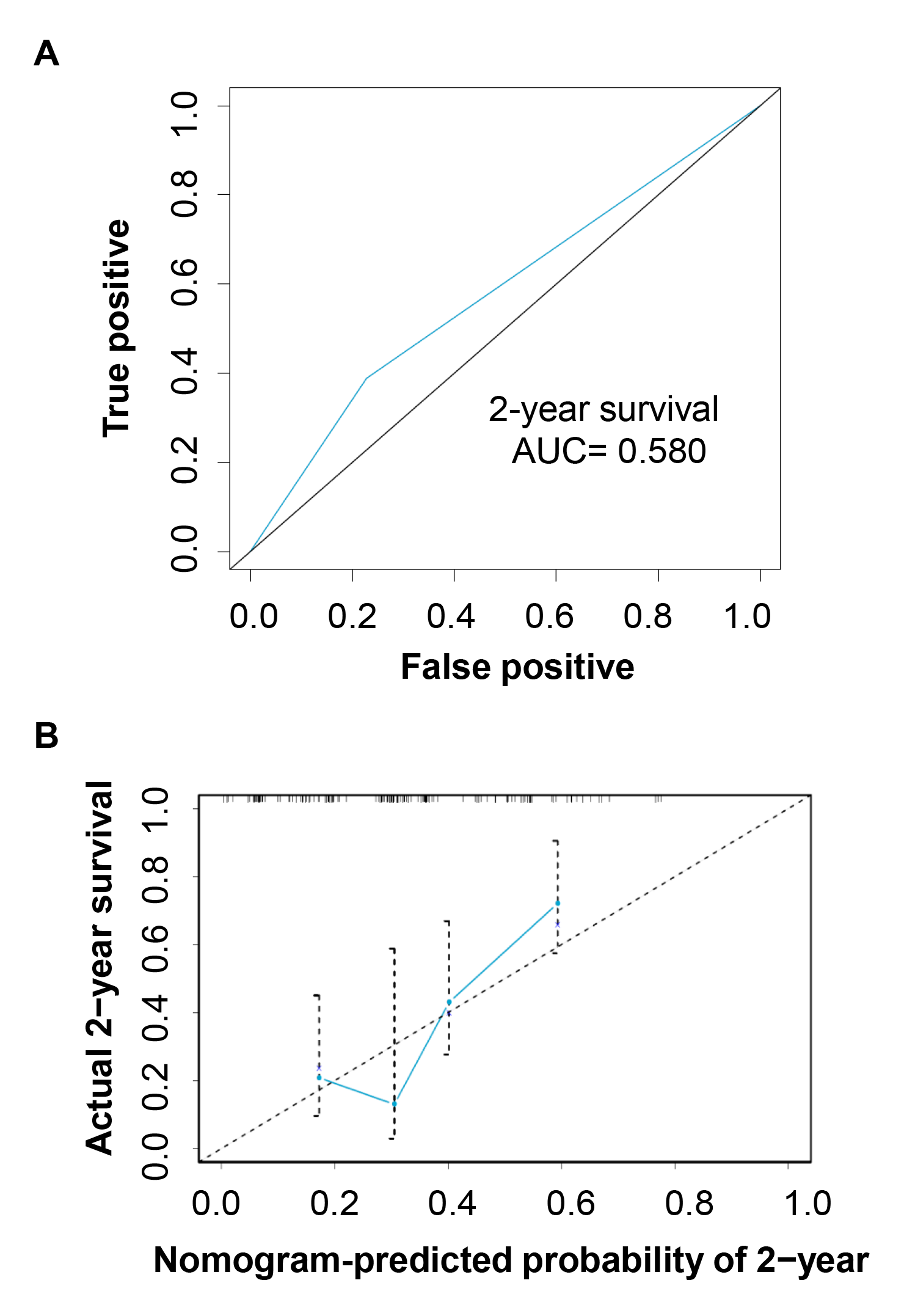

Supplement: Supplementary file 1 — Fig S1 [file CAM4-10-6218-s001.tif]

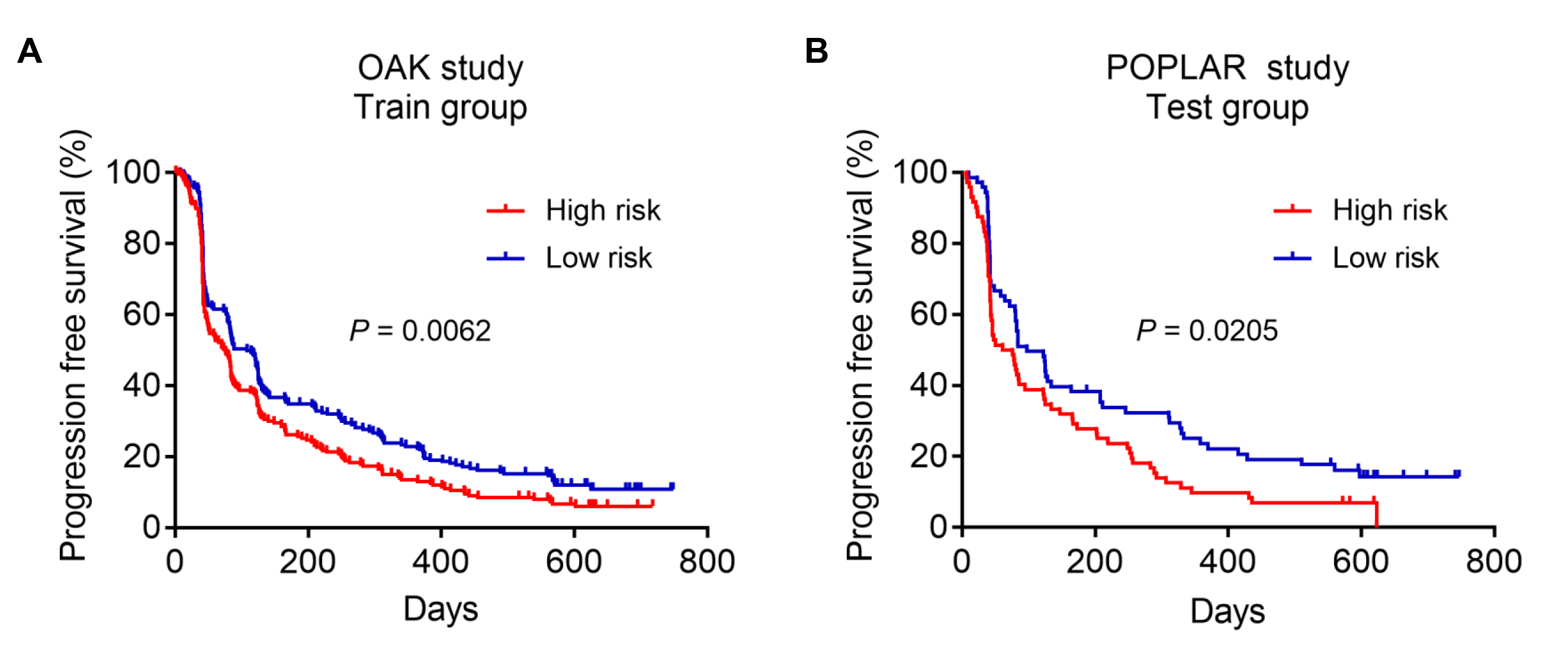

Supplement: Supplementary file 2 — Fig S2 [file CAM4-10-6218-s002.tif]

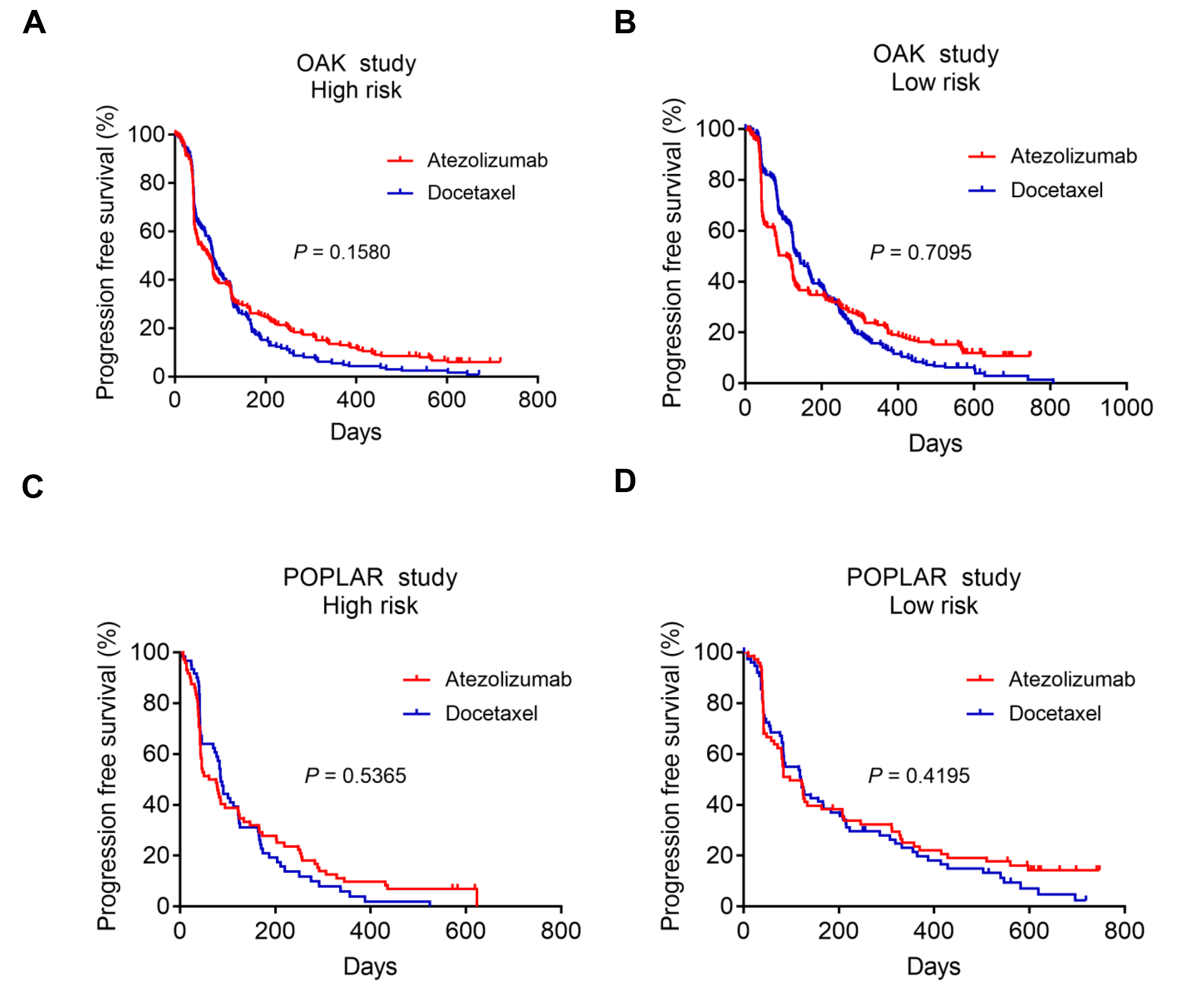

Supplement: Supplementary file 3 — Fig S3 [file CAM4-10-6218-s003.tif]
