# Supplementary material for: A clinical variable‐based nomogram could predict the survival for advanced NSCLC patients receiving second‐line atezolizumab
Source: Cancer Med. 2021 Jul 31;10(18):6218–26. doi: 10.1002/cam4.4160 (PMC8446569; doi:10.1002/cam4.4160)
Supplement: Supplementary file 4 — Data S1 [file CAM4-10-6218-s004.docx]

**Supplement Table 1.** **Baseline clinicopathological characteristics of 424 NSCLC patients treated with atezolizumab from OAK cohort in high- and low-risk group.**

| **Variables** | **High-risk group**  **(n =210)** | **Low-risk group**  **(n =214)** |
| --- | --- | --- |
| **Race** |  |  |
| White | 163 (77.6) | 139 (65.0) |
| Asian | 23 (11.0) | 62 (29.0) |
| Others | 24 (11.4) | 13 (6.0) |
| **Sex** |  |  |
| Male | 154 (73.3) | 106 (49.5) |
| Female | 56 (26.7) | 108 (50.5) |
| **Histology** |  |  |
| Non-squamous | 121 (57.6) | 192 (89.7) |
| Squamous | 89 (42.4) | 22 (10.3) |
| **ECOG PS** |  |  |
| 0 | 25 (36.6) | 130 (60.7) |
| 1 | 185 (63.4) | 84 (39.3) |
| **blSLD (mm)** |  |  |
| ≤67 | 52 (24.8) | 162 (75.7) |
| > 67 | 158 (75.2) | 52 (24.3) |
| **Metastatic sites** |  |  |
| 1-3 | 113 (53.8) | 186 (86.9) |
| >3 | 97 (46.2) | 28 (13.1) |

| **Variables** | **High-risk group**  **(n = 72)** | **Low-risk group**  **(n = 72)** |
| --- | --- | --- |
| **Race** |  |  |
| White | 58 (80.6) | 52 (72.2) |
| Asian | 8 (11.1) | 15 (20.8) |
| Others | 6 (8.3) | 5 (7.0) |
| **Sex** |  |  |
| Male | 57 (79.2) | 36 (50.0) |
| Female | 15 (20.8) | 36 (50.0) |
| **Histology** |  |  |
| Non-squamous | 38 (52.8) | 57 (79.2) |
| Squamous | 34 (47.2) | 15 (20.8) |
| **ECOG PS** |  |  |
| 0 | 6 (8.3) | 42 (58.3) |
| 1 | 66 (91.7) | 30 (41.7) |
| **blSLD (mm)** |  |  |
| ≤67 | 56 (77.8) | 49 (68.1) |
| > 67 | 16 (22.2) | 23 (31.9) |
| **Metastatic sites** |  |  |
| 1-3 | 37 (51.4) | 66 (91.7) |
| >3 | 35 (48.6) | 6 (8.3) |

**Supplement Table 2.** **Baseline clinicopathological characteristics of 144 NSCLC patients treated with atezolizumab from POPLAR cohort in high- and low- risk groups.**

**Supplement Table 3.** **Baseline clinicopathological characteristics of 425 NSCLC patients treated with docetaxel from OAK cohort in high- and low-risk group.**

| **Variables** | **High-risk group**  **(n = 200)** | **Low-risk group**  **(n = 225)** |
| --- | --- | --- |
| **Race** |  |  |
| White | 155 (77.5) | 141 (62.7) |
| Asian | 31 (15.5) | 64 (28.4) |
| Others | 14 (7.0) | 20 (8.9) |
| **Sex** |  |  |
| Male | 130 (65.0) | 129 (57.3) |
| Female | 70 (35.0) | 96 (42.7) |
| **Histology** |  |  |
| Non-squamous | 118 (59.0) | 197 (87.6) |
| Squamous | 82 (41.0) | 28 (12.4) |
| **ECOG PS** |  |  |
| 0 | 5 (2.5) | 155 (68.9) |
| 1 | 195 (97.5) | 70 (31.1) |
| **blSLD (mm)** |  |  |
| ≤67 | 50 (25.0) | 168 (74.7) |
| > 67 | 150 (75.0) | 57 (25.3) |
| **Metastatic sites** |  |  |
| 1-3 | 111 (55.5) | 191 (84.9) |
| >3 | 89 (44.5) | 34 (15.1) |

**Supplement Table 4.** **Baseline clinicopathological characteristics of 143 NSCLC patients treated with docetaxel from POPLAR cohort in high- and low-risk group.**

| **Variables** | **High-risk group**  **(n = 50)** | **Low-risk group**  **(n = 93)** |
| --- | --- | --- |
| **Race** |  |  |
| White | 40 (80.0) | 76 (81.7) |
| Asian | 7 (14.0) | 6 (6.5) |
| Others | 3 (6.0) | 11 (11.8) |
| **Sex** |  |  |
| Male | 28 (56.0) | 48 (51.6) |
| Female | 22 (44.0) | 45 (48.4) |
| **Histology** |  |  |
| Non-squamous | 36 (72.0) | 59 (63.4) |
| Squamous | 14 (28.0) | 34 (36.6) |
| **ECOG PS** |  |  |
| 0 | 10 (20.0) | 36 (38.7) |
| 1 | 40 (80.0) | 57 (61.3) |
| **blSLD (mm)** |  |  |
| ≤67 | 34 (68.0) | 48 (51.6) |
| > 67 | 16 (32.0) | 45 (48.4) |
| **Metastatic sites** |  |  |
| 1-3 | 0 (0.0) | 93 (100.0) |
| >3 | 50 (100.0) | 0 (0.0) |

ECOG, Eastern Cooperative Oncology Group; PS, performance status; blSLD, baseline sum of the longest diameters
